# Increased Von Willebrand factor, decreased ADAMTS13 and thrombocytopenia in melioidosis

**DOI:** 10.1371/journal.pntd.0005468

**Published:** 2017-03-15

**Authors:** Emma Birnie, Gavin C. K. W. Koh, Ester C. Löwenberg, Joost C. M. Meijers, Rapeephan R. Maude, Nicholas P. J. Day, Sharon J. Peacock, Tom van der Poll, W. Joost Wiersinga

**Affiliations:** 1 Center for Experimental and Molecular Medicine, Division of Infectious Diseases, Academic Medical Center, Amsterdam, The Netherlands; 2 Faculty of Tropical Medicine, Mahidol University, Bangkok, Thailand; 3 Department of Medicine, University of Cambridge, Addenbrooke’s Hospital, Cambridge, United Kindom; 4 Department of Infection and Tropical Medicine, Heartlands Hospital, Birmingham, United Kindom; 5 Department of Experimental Vascular Medicine, Academic Medical Center, Amsterdam, The Netherlands; 6 Department of Plasma Proteins, Sanquin Research, Amsterdam, The Netherlands; 7 The Centre for Clinical Vaccinology and Tropical Medicine, Nuffield Department of Clinical Medicine, University of Oxford, Churchill Hospital, Oxford, United Kindom; University of Liverpool, UNITED KINGDOM

## Abstract

**Background:**

Melioidosis, caused by bioterror treat agent *Burkholderia pseudomallei*, is an important cause of community-acquired Gram-negative sepsis in Southeast Asia and Northern Australia. New insights into the pathogenesis of melioidosis may help improve treatment and decrease mortality rates from this dreadful disease. We hypothesized that changes in Von Willebrand factor (VWF) function should occur in melioidosis, based on the presence of endothelial stimulation by endotoxin, pro-inflammatory cytokines and thrombin in melioidosis, and investigated whether this impacted on outcome.

**Methods/Principal findings:**

We recruited 52 controls and 34 culture-confirmed melioidosis patients at Sappasithiprasong Hospital in Ubon Ratchathani, Thailand. All subjects were diabetic. Platelet counts in melioidosis patients were lower compared to controls (*p* = 0.0001) and correlated with mortality (*p* = 0.02). VWF antigen levels were higher in patients (geometric mean, 478 U/dl) compared to controls (166 U/dL, *p*<0.0001). The high levels of VWF in melioidosis appeared to be due to increased endothelial stimulation (VWF propeptide levels were elevated, *p*<0.0001) and reduced clearance (ADAMTS13 reduction, *p<*0.0001). However, VWF antigen levels did not correlate with platelet counts implying that thrombocytopenia in acute melioidosis has an alternative cause.

**Conclusions/Significance:**

Thrombocytopenia is a key feature of melioidosis and is correlated with mortality. Additionally, excess VWF and ADAMTS13 deficiency are features of acute melioidosis, but are not the primary drivers of thrombocytopenia in melioidosis. Further studies on the role of thrombocytopenia in *B*. *pseudomallei* infection are needed.

## Author summary

Melioidosis, caused by bioterror threat agent *Burkholderia pseudomallei*, is an important cause of community-acquired sepsis in Southeast Asia and Northern Australia. Recently, it has been predicted that the annual burden of melioidosis is much higher than previously thought, with 165.000 human cases from which 89.000 patients die worldwide. Melioidosis has a mortality up to 40% despite appropriate antibiotic treatment and there is currently no vaccine available. Therefore, it is of importance to better understand the pathogenesis of this debilitating disease. There is extensive cross talk between the innate immune system and blood coagulation, which contributes to the host defense against invading bacteria. One of the hallmark features of melioidosis is extensive abnormalities in the coagulation system. Therefore, we hypothesized that, since endothelial stimulation by endotoxin, pro-inflammatory cytokines and thrombin all occur in melioidosis, these would result in derangements of Von Willebrand factor (a protein involved in hemostasis and platelet aggregation). In a cohort of culture-confirmed patients with severe melioidosis, we found that thrombocytopenia is a key feature of melioidosis and is correlated with mortality. Additionally, our study showed that excess VWF and ADAMTS13 deficiency are features of acute melioidosis, but are not the primary drivers of thrombocytopenia in melioidosis.

## Introduction

The soil-dwelling intracellular bacterium *Burkholderia pseudomallei* is an important cause of community-acquired Gram-negative sepsis in Southeast Asia and Northern Australia [1, 2], and the causative agent of melioidosis. Recently, it has been predicted that the annual burden of melioidosis is much higher than previously thought, with 165.000 human cases from which 89.000 patients die worldwide [3]. Over half of patients are bacteraemic at presentation [1] and despite appropriate antibiotic therapy, melioidosis has a mortality rate of 14–40% [1]. There is currently no vaccine available. The high mortality rate and the emerging antibiotic resistance of *B*. *pseudomallei* [4] highlights the need to better understand the pathogenesis of melioidosis.

The interaction between innate immunity and blood coagulation contribute to the host defense against bacteria, in attempt to contain the infectious agent at the site of infection and prevent further dissemination [5, 6]. Ample evidence has shown that severe melioidosis is characterized by strong activation of the coagulation system (as reflected by high plasma levels of soluble tissue factor, the prothrombin fragment F1+2 and thrombin–antithrombin complexes), a downregulation of anticoagulant pathways (as shown by decreased levels of protein C, protein S, and antithrombin) and both activation and inhibition of fibrinolysis (as reflected by elevated concentrations of tissue-type plasminogen activator (tPA), plasminogen activator inhibitor type 1 and plasmin-a2-antiplasmin complexes (PAPc)) [7–11]. Concurrently, a consumption of coagulation factors results in a prolonged prothrombin time and activated partial thromboplastin time [8].

Von Willebrand factor (VWF), a circulating multimeric glycoprotein, is intimately involved in hemostasis and platelet activation and aggregation [5, 12]. VWF excess is therefore associated with platelet consumption and thrombocytopenia [13–15]. VWF is constitutively expressed by endothelial cells and stored in Weibel-Palade bodies, but can also be released following stimulation by endotoxin, cytokines or thrombin and is consequently detectable in its native, ultralarge isoform (ulVWF). VWF dysregulation might lead to microvascular thrombosis [16].

ADAMTS13 (A Disintegrin and Metalloproteinase with a Thrombospondin type-1 motif member 13), is a plasma protease primarily synthesized and secreted from hepatic stellate cells (HSCs) [17] and is known to be the main regulator of VWF activity by cleavage of the A2 domain within shear activated VWF [13, 17]. ADAMST13 plasma activity below 10% (<5% depending on the assay used) goes along with thrombotic microangiopathies and bleedings known as thrombotic thrombocytopenic purpura (TTP) [18].

VWF, ADAMTS13 and platelets have been suggested as possible biomarkers for microangiopathic diseases such as sepsis [19, 20]. We hypothesize that since endothelial stimulation by endotoxin, pro-inflammatory cytokines and thrombin all occur in melioidosis [8, 21, 22], these would result in derangements of VWF in the host defense against septic melioidosis. First of all, we found that thrombocytopenia is a feature of melioidosis and is correlated with mortality. Additionally, study results showed that excess VWF and ADAMTS13 deficiency are features of acute melioidosis, but are not the primary drivers of thrombocytopenia in melioidosis.

## Methods

### Ethics statement

The study was approved by the Ethics Committee of the Faculty of Tropical Medicine, Mahidol University (MUTM 2008-001-01) and the Oxford Tropical Research Ethics Committee (OXTREC 018–07). Written informed consent was obtained from all subjects or next-of-kin by a native Thai speaker. All procedures were performed in accordance with the Helsinki Declaration of 1975 (revised 1989).

### Study population

Eligible patients were aged 18–75 years, had culture-proven melioidosis, had received active antimicrobial chemotherapy for less than 48 hours (ceftazidime, co-amoxiclav, meropenem or imipenem), and had ≥ two out of four criteria for systemic inflammatory response syndrome (SIRS) [23]. This cohort has been previously described [9]. Controls were seen once and not followed-up; patients were seen daily until death or discharge and then seen at the first follow-up outpatient clinic.

Plasma samples were collected at admission, seven days after and at the first outpatient clinic ≥28 days after discharge. We excluded pregnant women, and patients on anticoagulants or immunosuppressive therapy.

Melioidosis patients were classified as diabetic if they had a diagnosis of diabetes prior to the onset of illness or an admission HbA_1c_ ≥7.8% [24]. The study was restricted to patients with diabetes only for the following reasons: diabetes itself has effects on coagulation, the majority of patients with melioidosis have diabetes, and we were not interested in the effect of diabetes on coagulation during melioidosis, which has been investigated extensively elsewhere [9]. Melioidosis patients who do not have diabetes as a risk factor commonly have other risk factors such as corticosteroid immunosuppression, cancer, renal failure, and so forth [1], many of which are themselves associated with endothelial stimulation and abnormalities of coagulation, making it very difficult to identify an appropriate control group. Restriction is a well-established design technique in epidemiology [25].

### Assays

Blood samples were collected once only from controls, and up to three times from patients (at recruitment, seven days later and at the first follow-up clinic >28 days from discharge). No samples were collected at any other time points. HbA_1C_ was measured by high performance liquid chromatography (Bio-Rad D-10, Bio-Rad Laboratories, Hercules, California). Hemoglobin (Hb), white blood cell count (WBC), neutrophils, lymphocytes, thrombocytes, creatinine, alanine aminotransferase (ALT), aspartate aminotransferase (AST), alkaline phosphatase (ALP) and bilirubin were routinely available as part of the initial assessment of all participants. Blood for coagulation assays was collected in citrated tubes (Becton-Dickinson Vacutainer 369714) and plasma was removed after centrifugation at 1000 ×*g* for 10 minutes. The plasma was stored at –70°C pending assay in The Netherlands. VWF antigen (Dako, Glostrup, Denmark) and VWF propeptide (Sanquin, Amsterdam, The Netherlands) were assayed by enzyme-linked immunoassay as described previously [15]. ADAMTS13 levels and prothrombin time (PT) were measured on an automated blood coagulation analyzer (BCS XP, Siemens Healthcare Diagnostics, Marburg, Germany) [26, 27]. Fibrinogen levels were derived from the change in optical signal in the PT. VWF antigen, VWF propeptide and ADAMTS13 results were expressed in U/dL, where 1 unit is the activity of 1 ml of pooled normal plasma. PT was expressed in seconds and fibrinogen levels were expressed as g/L.

### Statistical analysis

Statistical analyses were performed and plots generated on GraphPad Prism 5.0b (Graphad Software, San Diego, CA). Quantile-quantile plots were checked for normality and to select appropriate transformations. The distribution of age, PT, and ADAMTS13 levels were Gaussian. HbA1c levels of VWF antigen and propeptide were log-normal. An inverse square-root transform was applied to fibrinogen. Hb, WBC, neutrophils, lymphocytes, thrombocytes, creatinine, ALT, AST, ALP and bilirubin could not be transformed to Gaussian and were therefore analyzed non-parametrically. Other continuous variables were compared using the Student t-test with Welch’s modification applied when appropriate. Thrombocytopenia was defined as platelet count <150 × 10^9^/l. Categorical data were compared by Fisher’s exact test. Strength of correlation was reported using Pearson’s coefficient. *P*-values were interpreted as recommended by Stern and Davey Smith [28].

## Results

### Patient characteristics

We recruited 52 controls and 34 culture-confirmed melioidosis patients at Sappasithiprasong Hospital in Ubon Ratchathani, Thailand. All patients were septic (see inclusion criteria) and had diabetes. Controls were, therefore, otherwise healthy diabetics attending a routine out-patient diabetes clinic. Their baseline characteristics are presented in Table 1 and their laboratory findings are depicted in S1 Table. This cohort has been previously described elsewhere [9]. In the melioidosis group, 12 patients died (35%) before the first follow up (≥28 days after enrolment).

**Table 1:** Baseline characteristics from 52 controls and 34 melioidosis patients. Age, glucose and HbA_1_c are reported as mean (95% confidence interval). Male sex and mortality are reported as percentages (no).

	Controls (n = 52)	Melioidosis patients (n = 34)
**Age, years**	57.5 (54.1–60.9)	52.9 (49.8–56.0)
**Male sex**	34.6% (18/52)	61.8% (21/34)
**HbA**_**1C, %**_	8.2% (7.8–8.5)	10.6% (9.6–11.7)
**Mortality**	_	35.2% (12/34)

### Melioidosis is associated with thrombocytopenia

**Fig 1:**
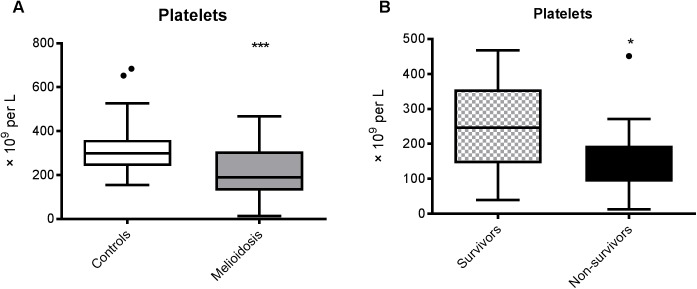
**Thrombocytopenia is a feature of acute melioidosis (A) and correlates with mortality (B).** The data from 34 melioidosis patients (of whom 12 died) and 52 controls are presented as box plots with Tukey whiskers showing the smallest observation, lower quartile, median, upper quartile and largest observation. ***P <0.001 for the difference between patients and controls; *P <0.05 for the difference between survivors (n = 22) and non-survivors (n = 12) (Student’s t-test).

An important function of VWF is to mediate platelet-platelet interactions and platelet adhesion to sub-endothelial collagen and is associated with platelet consumption and thrombocytopenia [13–15]. The median platelet count in patients with melioidosis was 189 × 10^9^/l compared to 299 × 10^9^/l in controls (*p* = 0.0001, Fig 1A). There were 14 melioidosis patients (41%) with thrombocytopenia (defined as a platelet count <150 × 10^9^/l) and no cases of thrombocytopenia among controls (*p*<0.0001). Among patients, the lowest admission platelet count observed was 13 × 10^9^/l. Platelet counts were lower in non-survivors (median 138 × 10^9^/l) compared to survivors (247 × 10^9^/l, *p* = 0.02, Fig 1B). Of the 12 patients who died, eight (67%) had thrombocytopenia compared to six of the survivors (27%, *p* = 0.04).

### VWF levels are elevated in melioidosis

**Fig 2:**
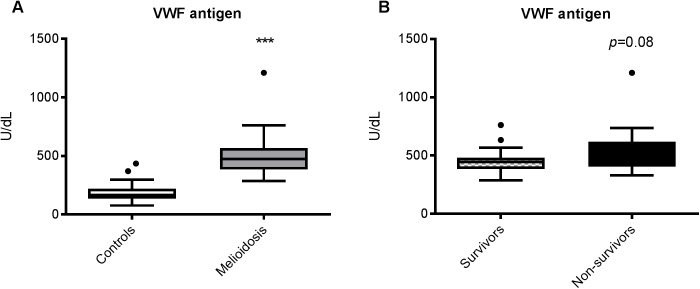
**VWF antigen levels are also elevated in melioidosis (A), but do not correlate with mortality (B).** VWF = von Willebrand factor. The data from 34 melioidosis patients (of whom 12 died) and 52 controls are presented as box plots with Tukey whiskers showing the smallest observation, lower quartile, median, upper quartile and largest observation. ***P <0.001 for the difference between patients and controls; *P* = 0.08 for the difference between survivors (n = 22) and non-survivors (n = 12) (Student’s t-test).

Having seen thrombocytopenia in acute melioidosis, we predicted that this would be driven by high levels of circulating VWF. We observed that VWF antigen levels were higher in patients (geometric mean, 478 U/dl) compared to controls (166 U/dL, *p*<0.0001, Fig 2A). However, the level of VWF antigen at recruitment was not associated with mortality (geometric mean 445 U/dL in survivors versus 540 U/dL in non-survivors, *p* = 0.08, Fig 2B).

### Excess circulating VWF in melioidosis is driven by excess secretion

**Fig 3:**
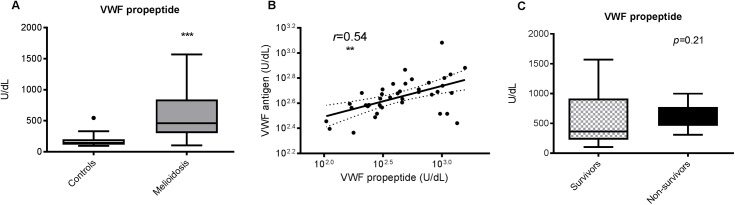
**VWF propeptide concentrations are elevated in melioidosis (A) and correlate with VWF antigen levels (B), but do not correlate with mortality (C).** VWF = von Willebrand factor. The data from 34 melioidosis patients (of whom 12 died) and 52 controls are presented as box plots with Tukey whiskers showing the smallest observation, lower quartile, median, upper quartile and largest observation. ***P <0.001 for the difference between patients and controls; (Student’s t-test); *P* = 0.21 for the difference between survivors (n = 22) and non-survivors (n = 12). For the scatter plot, each dot represents a single study subject from the patient group only (n = 34); the correlation coefficient and *P <0.05 reported are for Pearson’s *r*. The corresponding regression line for the scatter plot is drawn in bold, with the 95% confidence interval for the regression line marked by interrupted lines.

Next, we looked at whether the excess VWF antigen might be explained by increased secretion. VWF propeptide is a marker for recent secretion of VWF from the Weibel-Palade bodies (WBD) of endothelial cells and the dense granules of platelets [29]. We found VWF propeptide concentrations were higher in patients (460 U/dL) compared to controls (159 U/dL, *p*<0.0001, Fig 3A). Furthermore, VWF propeptide levels correlated well with VWF antigen levels (Pearson’s *r* = 0.54, *p* = 0.003, Fig 3B), supporting our hypothesis that the excess in circulating VWF was due to excess secretion of VWF. VWF propeptide concentration and survival were not correlated, and the range of values obtained in non-survivors fell within the range obtained for survivors (*p* = 0.21, Fig 3C).

### Excess circulating VWF antigen is also driven by reduced ADAMTS13

**Fig 4:**
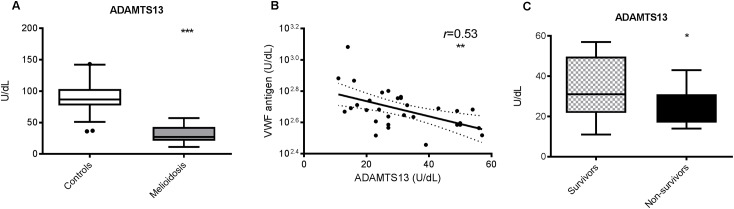
**ADAMTS13 levels are depressed in melioidosis patients (A) and are correlated with VWF antigen levels (B), however only weak correlation between ADAMS13 and mortality (C).** ADAMTS13 = A Disintegrin and Metalloproteinase with a Thrombospondin type-1 motif member 13. The data from 34 melioidosis patients (of whom 12 died) and 52 controls are presented as box plots with Tukey whiskers showing the smallest observation, lower quartile, median, upper quartile and largest observation. ***P <0.001 for the difference between patients and controls; (Student’s t-test); *P* = 0.21 for the difference between survivors (n = 22) and non-survivors (n = 12). For the scatter plot, each dot represents a single study subject from the patient group only (n = 34); the correlation coefficient and *P <0.05 reported are for Pearson’s *r*. The corresponding regression line for the scatter plot is drawn in bold, with the 95% confidence interval for the regression line marked by interrupted lines.

ADAMTS13 is a metalloprotease secreted by the liver known as VWF cleaving protease [14, 17]. Deficiencies of ADAMTS13 results in the accumulation of VWF in the circulation and, consequently, thrombocytopenia [13, 14]. Previous studies have found an association between sepsis and reduced levels of ADAMTS13 [30, 31]. The mean ADAMTS13 concentration was 31 U/dL in patients and 90 U/dL in controls (*p*<0.0001, Fig 4A). ADAMTS13 levels and VWF antigen were negatively correlated (*r =* 0.53, *p* = 0.002, Fig 4B), which supports our hypothesis that decreased levels of ADAMTS13 contribute to high concentrations of VWF in melioidosis. However, there was only weak evidence for an inverse correlation between ADAMTS13 deficiency and mortality in melioidosis (*p* = 0.05, Fig 4C). Although the mean ADAMTS13 level in non-survivors (26 U/dL) was lower than that in survivors (34 U/dL), the range of values obtained in non-survivors (14 to 43 U/dL) fell entirely within the range of values obtained in survivors (11 to 57 U/dL).

### VWF is not the main driver of thrombocytopenia in melioidosis

Thrombocytopenia was a feature of acute melioidosis and correlates with mortality. We also found high levels of VWF in melioidosis, which were both explained by increased secretion of pre-formed VWF and by reduced clearance of VWF. However, if VWF were the main driver of thrombocytopenia in melioidosis, then it is surprising that VWF antigen, VWF propeptide and ADAMTS13 levels do not correlate with mortality. We therefore re-examined the relationship between VWF antigen levels and platelet count, and found that although both were deranged in melioidosis, their levels were not correlated (*r* = 0.28, *p* = 0.12, S1 Fig).

### Platelet counts, VWF antigen and ADAMTS13 return to normal following recovery from melioidosis

In those patients who survived, a follow-up sample was taken seven days following enrollment and at the first follow-up clinic (≥28 days after discharge). In all patients, perturbations of platelet counts as well as abnormalities in levels of VWF antigen, VWF propeptide and ADAMTS13 all resolved completely following recovery from melioidosis (S2A–S2D Fig).

## Discussion

In the current study, we investigated derangements of VWF in the host defense against septic melioidosis. Thrombocytopenia has been observed incidentally in previous cases of melioidosis [32, 33] and so we first sought evidence of thrombocytopenia in melioidosis, since platelet counts are routinely available as part of the initial assessment of all sepsis patients. We found that thrombocytopenia is a feature of sepsis caused by *B*. *pseudomallei* and is correlated with mortality.

The concept that platelets are the chief cellular effector of hemostasis is well established [34], however in very recent years we and others have showed in two preclinical studies that platelets also function as key effector players in the host response against bacterial infections [35, 36]. Of note, mice treated with the platelet depleting antibody (α-GpIbα) had a strongly impaired host response when intranasally challenged with Gram-negative bacteria leading to increased bacterial growth and a decreased survival [35]. Additionally, a recent clinical study which included a heterogeneous group of 913 consecutive patients with sepsis, blood microarray analysis revealed a distinct gene expression pattern in sepsis patients, low platelet counts, showing reduced signaling in leukocyte adhesion and diapedesis and increased complement signaling [37]. Platelets interact with the innate immune system through different mechanisms; direct bacterial killing, immunothrombosis, recruitment of neutrophils, and by potentiating effects such as neutrophil extracellular traps (NETs) production [5, 38]. NETs form a central role in the host response against invading pathogens, and entrap and kill bacteria. We previously showed that melioidosis patients had increased levels of NET-related components and that NETs have antibacterial activity against *B*. *pseudomallei* [39]. However, NET formation did not protect against bacterial dissemination or inflammation in a murine *B*. *pseudomallei*-induced sepsis model [39].

In septic patients, the development of thrombocytopenia is secondary to various mechanism [40]; platelets are activated and bound to endothelium, resulting in sequestration and destruction [41]. It remains unclear whether reduced platelet counts lead directly to adverse clinical outcome in sepsis, or whether they are simply a biomarker for disease severity at presentation [42]. We show that thrombocytopenia is related with progression to death in melioidosis which is probably associated with a more disturbed host response [37]. However, further clinical and animal studies on the exact role of platelets and platelet neutrophil interactions in the host response against *B*. *pseudomallei* infection are needed.

In our study, VWF antigen levels were higher in melioidosis patients compared to controls, however not correlated with mortality. We showed that excess circulating VWF in melioidosis is correlated with excess secretion and reduced ADAMST13. Our finding that ADAMST13 activity is decreased in melioidosis is consistent with the wider sepsis literature which finds that ADAMST13 deficiency is a common feature of severe sepsis both in adults [30] and in children [20]. This decline of ADAMST13 may be explained by the presence of proinflammatory cytokines in septic patients [22], which suppress mRNA transcription encoding ADAMTS13 [43]. ADAMST13 was decreased in non-surviving patients potentially reflecting the decrease of proteolytic activity of this enzyme in sepsis [31, 44]. However, literature contains conflicting data on the utility of VWF and ADAMTS13 as predictors of outcome in sepsis [16]. In a single-center observational study of 40 patients conducted as part of a larger randomized-controlled trial of C1-inhitbitor supplementation in all-cause sepsis they show that ADAMST13 levels were lower and VWF antigen levels were higher in severe sepsis or septic shock [31]. Neither ADAMST13 nor VWF parameters correlated with outcome. In our study, the range of ADAMST13 values felt within the normal range. We therefore, can conclude that ADAMTS13 is of no prognostic value in melioidosis. Moreover, as the link between ADAMST13 levels and VWF antigen is not as strong and the correlation with mortality is weak, novel strategies for the adjunctive treatment of sepsis such as ADAMTS13 supplementation that have been proposed for the management of all-cause sepsis [44] are also less likely to be beneficial in melioidosis.

Interestingly, there was no correlation between VWF and thrombocytopenia, suggesting that excess VWF is not the primary driver of thrombocytopenia in melioidosis. We note, incidentally, that other additional mechanisms may contribute to the accumulation of VWF during sepsis: for example, oxidative modification of VWF in sepsis has been shown to prevent cleavage of VWF by ADAMST13 [45, 46]. Another potential driver of thrombocytopenia includes diffuse intravascular coagulation (DIC) [47]. Activation of the coagulation pathway may be deleterious when the triggered blood coagulation is insufficiently controlled. This could lead to DIC and microvascular thrombosis. Reduced level of ADAMTS13, as we showed in this study, has been reported in DIC due to severe sepsis [30]. However, the median DIC score from this melioidosis cohort, as calculated using the International Society on Thrombosis and Haemostasis (ISTH) standardized method was 3 (2–4), indicating that DIC is not overt [9, 48]. More studies focusing on DIC in melioidosis patients are needed.

Additionally, hemophagocytosis, a life-threatening condition of excessive immune activation, has been postulated to drive sepsis-related thrombocytopenia. Proliferation of macrophages, leading to uncontrolled phagocytosis of platelets, erythrocytes and lymphocytes characterizes the macrophage activation syndrome (MAS) [49, 50]. It could very well be that a significant portion of our patients have MAS, which can be defined as having five of the following eight features: fever, splenomegaly, peripheral blood cytopenia, hypertriglyceridemia and/or hyperfibrinogenemia, hemophagocytosis in bone marrow, spleen, lymph node of liver, diminished NK cell activity, high ferritin levels and elevated soluble CD35 [51]. Unfortunately, most of these markers are not available for our patients. This is an interesting area to further explore. Patients with sepsis associated MAS might benefit from interleukin-1 receptor blockade [50]. In this respect, it is of interest that both the administration of anti-IL-1ra as well as anti-IL-1b protects against experimental melioidosis [52, 53].

To the best of our knowledge, this work is the first that studies the impact of VWF, ADAMST13 and platelets in patients with melioidosis. However, study limitations need to be considered. First, we only included patients with diabetes in this study. We made this decision, because diabetes itself is associated with abnormalities of coagulation, anticoagulation and fibrinolysis [54]. In addition, the majority of patients with melioidosis have diabetes and we were not interested in the effect of diabetes on coagulation during melioidosis, because this has been investigated extensively elsewhere [9]. Second, the number of patients analyzed was restricted due to financial constraints for the assays. Third, although to the best of our knowledge no intersex differences in VWF and ADAMTS13 levels have been described, it should be mentioned that the male/female ratio of controls differs from patients.

### Conclusions

In conclusion, thrombocytopenia is a feature of sepsis caused by *B*. *pseudomallei* and is correlated with mortality. Excess VWF is a feature of acute melioidosis and is likely driven by both by increased secretion of VWF propeptide in endothelium and by reduced clearance by ADAMTS13. However, the thrombocytopenia of melioidosis is likely not driven by excess VWF: other possible drivers include diffuse intravascular coagulation (DIC) and hemophagocytosis. In the past years, tremendous progress has been made toward our understandings of the protective roll of platelets in sepsis and the possibility in the use of thrombocytopenia as biomarker [35, 37]. More animal and human studies are necessary to understand the reason of thrombocytopenia in septic melioidosis patients and to translate this to clinical practice.

## Supporting information

S1 Checklist Strobe Checklist. (DOC)

S1 Table Summary of basic laboratory findings from 52 controls and 34 melioidosis patients. All values are reported as median with inter quartile ranges (IQR), except PT and fibrinogen which are reported as mean with confidence interval (CI). Values of PT and fibrinogen have been reported earlier [9]. Hb, hemoglobin; WBC, white blood cell count; ALT, alanine aminotransferase; AST, aspartate aminotransferase; ALP, alkaline phosphatase; PT, prothrombin time. *P*-values for the difference between patients and controls (Mann Whitney U test).
(DOCX)

S1 Fig VWF is not the main driver of thrombocytopenia in melioidosis. For the scatter plot; each dot represents a single study subject from the patient group only (n = 34); the correlation coefficient and *p*-value reported are for Pearson’s *r*. The corresponding regression line for each scatter plot is drawn in bold, with the 95% confidence interval for the regression line marked by interrupted lines.
(TIF)

S2 Fig In those patients who survived, the platelet count (A), VWF antigen level (B), VWF propeptide (C), and ADAMTS13 activity (D) all returned to normal following recovery.
VWF = Von Willebrand factor.
ADAMTS13 = A Disintegrin and Metalloproteinase with thrombospondin type 1 motif, member 13.
Data reported from melioidosis survivors (n = 24) are for admission (day zero), seven days after admission and at the first follow-up clinic (≥28 days after discharge). The grey shaded area represents the 5–95% quantiles for Thai diabetic controls. Abnormalities of platelets, ADAMTS13 and VWF parameters all normalize in those patients who survive melioidosis.
(TIF)
