# Correction: Increased Von Willebrand factor, decreased ADAMTS13 and thrombocytopenia in melioidosis

**DOI:** 10.1371/journal.pntd.0008722

**Published:** 2020-09-01

**Authors:** Emma Birnie, Gavin C. K. W. Koh, Ester C. Löwenberg, Joost C. M. Meijers, Rapeephan R. Maude, Nicholas P. J. Day, Sharon J. Peacock, Tom van der Poll, W. Joost Wiersinga

There is an error in the article XML causing the eighth author’s name to be indexed incorrectly. The name should be indexed as van der Poll T.
