# Supplementary material for: Increased Von Willebrand factor, decreased ADAMTS13 and thrombocytopenia in melioidosis
Source: PLoS Negl Trop Dis. 2017 Mar 15;11(3):e0005468. doi: 10.1371/journal.pntd.0005468 (PMC5376340; doi:10.1371/journal.pntd.0005468)
Supplement: S1 Table — All values are reported as median with inter quartile ranges (IQR), except PT and fibrinogen which are reported as mean with confidence interval (CI). Values of PT and fibrinogen have been reported earlier [9]. Hb, hemoglobin; WBC, white blood cell count; ALT, alanine aminotransferase; AST, aspartate aminotransferase; ALP, alkaline phosphatase; PT, prothrombin time. P-values for the difference between patients and controls (Mann Whitney U test). (DOCX) [file pntd.0005468.s002.docx]

|  | Controls (n=52) | Melioidosis patients (n=34) | *P*- values |
| --- | --- | --- | --- |
| Hb, g dL^-1^ | 12.5 (11.2-13.7) | 10.0 (8.5-11.1) | <0.0001 |
| WBC, x10^9^ L^-^ | 8.8 (7.2-10.2) | 10.2 (8.5-16.3) | 0.0066 |
| Neutrophils, % | 51 % (52-67) | 81 % (71-86) | <0.0001 |
| Lymphocytes, % | 27 % (22.5-32.5) | 12 % (8.8-17) | <0.0001 |
| Thrombocytes, x10^9^ L^-^ | 299 (241-360) | 189 (129-308) | <0.0001 |
| Serum creatinine, mg dL^-1^ | 0.9 (0.7-1.4) | 1.5 (0.8-2.6) | 0.0078 |
| ALT, U L^-1^ | 17 (12-31.5) | 50.5 (41.8-95.5) | <0.0001 |
| AST, U L^-1^ | 23.5 (19-26.5) | 87.5 (53.3-169.5) | <0.0001 |
| ALP, U L^-1^ | 83 (70-102.1) | 195.5 (150.5-448) | <0.0001 |
| Bilirubin, mg dL^-1^ | 0.7 (0.5-1.1) | 1.1 (0.4-4.0) | 0.1869 |
| PT, seconds | 11.5 (11.3-11.6) | 15.1 (14.3-15.9) | <0.001 |
| Fibrinogen, g L^-1^ | 8.2 (7.8-8.5) | 6.1 (5.3-7.1) | <0.001 |

**S1 Table. Summary of basic laboratory findings** **from 52 controls and 34 melioidosis patients.** All values are reported as median with inter quartile ranges (IQR), except PT and fibrinogen which are reported as mean with confidence interval (CI). Values of PT and fibrinogen have been reported earlier [9]. Hb, hemoglobin; WBC, white blood cell count; ALT, alanine aminotransferase; AST, aspartate aminotransferase; ALP, alkaline phosphatase; PT, prothrombin time. *P*-values for the difference between patients and controls (Mann Whitney U test).
