# Supplementary figures and images for: Increased Von Willebrand factor, decreased ADAMTS13 and thrombocytopenia in melioidosis
Source: PLoS Negl Trop Dis. 2017 Mar 15;11(3):e0005468. doi: 10.1371/journal.pntd.0005468 (PMC5376340; doi:10.1371/journal.pntd.0005468)

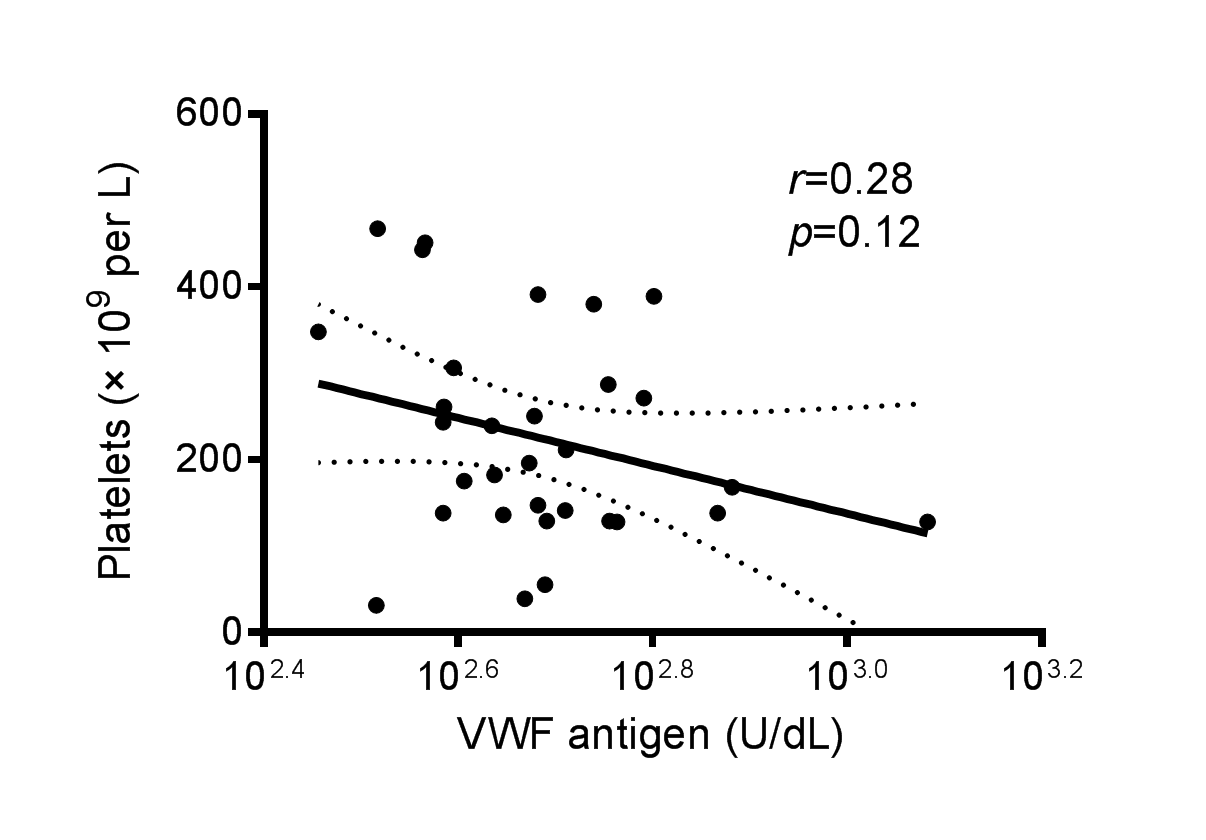

Supplement: S1 Fig — For the scatter plot; each dot represents a single study subject from the patient group only (n = 34); the correlation coefficient and p-value reported are for Pearson’s r. The corresponding regression line for each scatter plot is drawn in bold, with the 95% confidence interval for the regression line marked by interrupted lines. (TIF) [file pntd.0005468.s003.tif]

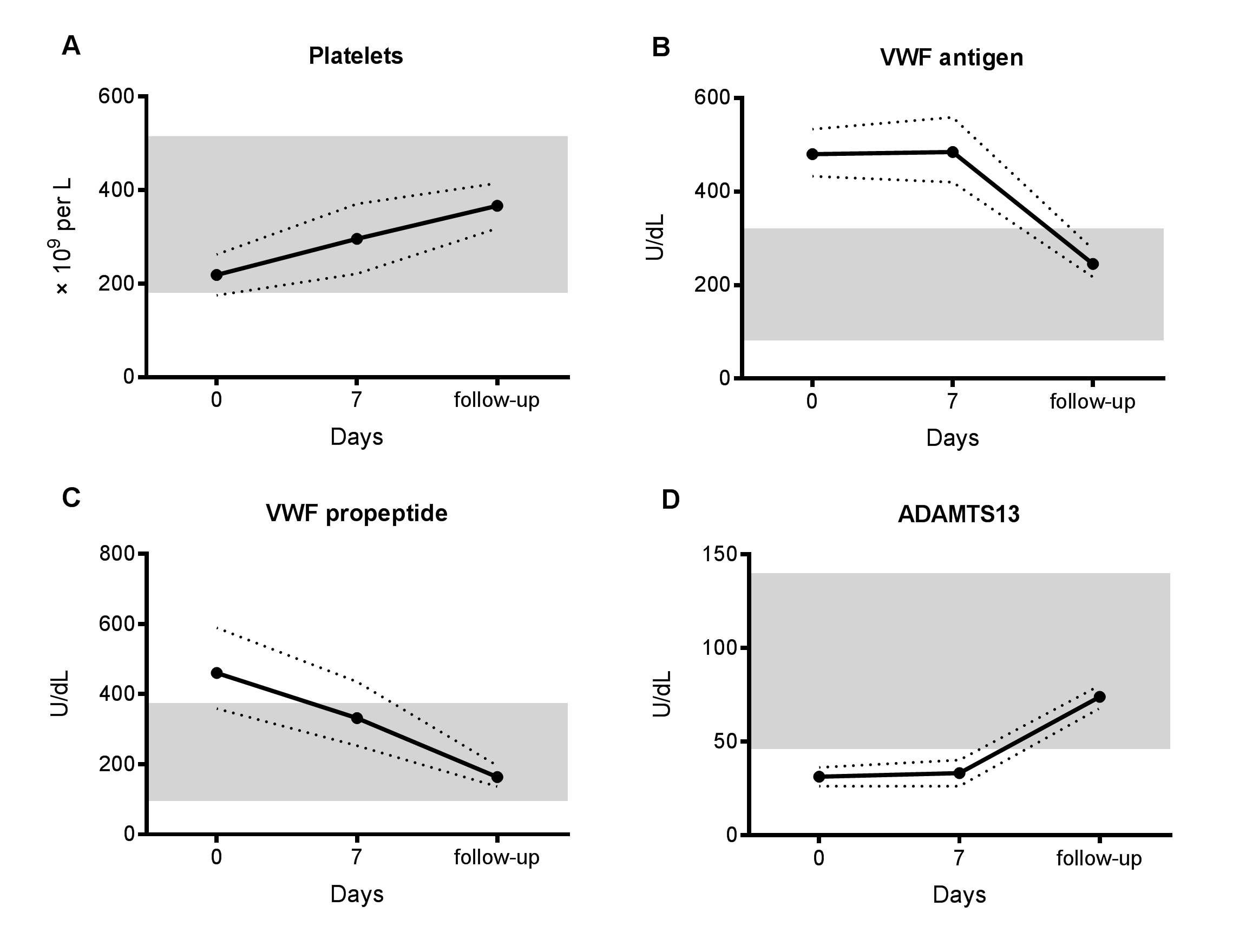

Supplement: S2 Fig — In those patients who survived, the platelet count (A), VWF antigen level (B), VWF propeptide (C), and ADAMTS13 activity (D) all returned to normal following recovery. VWF = Von Willebrand factor. ADAMTS13 = A Disintegrin and Metalloproteinase with thrombospondin type 1 motif, member 13. Data reported from melioidosis survivors (n = 24) are for admission (day zero), seven days after admission and at the first follow-up clinic (≥28 days after discharge). The grey shaded area represents the 5–95% quantiles for Thai diabetic controls. Abnormalities of platelets, ADAMTS13 and VWF parameters all normalize in those patients who survive melioidosis. (TIF) [file pntd.0005468.s004.tif]
